# Macrophage migration inhibitory factor is associated with mortality in cerebral malaria patients in India

**DOI:** 10.1186/1756-0500-2-36

**Published:** 2009-03-06

**Authors:** Vidhan Jain, Shannon McClintock, Avinash C Nagpal, Aditya P Dash, Jonathan K Stiles, Venkatachalam Udhayakumar, Neeru Singh, Naomi W Lucchi

**Affiliations:** 1 National Institute of Malaria Research, Regional Medical Research Center for Tribals, Indian Council of Medical Research, Jabalpur, India; 2 Malaria Branch, Division of Parasitic Diseases, National Center for Zoonotic Vector-Borne and Enteric Diseases, Coordinating Center for Infectious Diseases, Centers for Disease Control and Prevention, Atlanta, GA, USA; 3 Atlanta Research and Education Foundation, Decatur GA, USA; 4 Netaji Subash Chandra Bose Medical College, Jabalpur, India; 5 National Institute of Malaria Research, ICMR, New Delhi, India; 6 Morehouse School of Medicine, Atlanta, GA, USA

## Abstract

**Background:**

Macrophage migration inhibitory factor (MIF) is a multifunctional cytokine implicated in the pathogenesis of a number of human diseases including inflammatory neurological diseases. Its role in the pathogenesis of cerebral malaria is unknown. Cerebral malaria is a life-threatening complication of *falciparum *malaria with approximately 20%–30% of patients dying despite appropriate anti-malarial treatment. The reason for this cerebral malaria mortality is still unknown although host proinflammatory factors have been shown to be evidently important. The current study investigated the role of circulating MIF in the pathogenesis and outcomes of cerebral malaria.

**Findings:**

Three categories of subjects contributed to this study: healthy controls subjects, mild malaria patients, and cerebral malaria patients. The cerebral malaria patients were further grouped into cerebral malaria survivors and cerebral malaria non-survivors. MIF levels in the peripheral blood plasma, obtained at the time of enrollment, were measured using standard ELISA methods. In logistic regression on cerebral malaria patients, log MIF levels were found to be significantly associated with fatal outcome (odds ratio 4.0; 95%CI 1.6, 9.8; p = 0.003). In multinomial logistic regression log MIF levels were found to be significantly associated with patient category (p = 0.004).

**Conclusion:**

This study suggests that elevated levels of MIF in the peripheral blood of cerebral malaria patients may be associated with fatal outcomes.

## Background

Macrophage migration inhibitory factor (MIF) is a multifunctional cytokine whose role as an important regulator of immune and inflammatory responses in a number of human diseases, such as sepsis, rheumatoid arthritis, cancer and inflammatory neurological diseases, has been confirmed (reviewed in [1]). Its role in the pathogenesis of malaria has only begun to be investigated. The potential role of MIF in the pathogenesis of malaria anemia was evident from an experimental study using a mouse model in which high MIF levels were associated with malaria anemia [2]. However, most human studies conducted with African children have reported lower levels of MIF in malaria infected children compared to healthy asymptomatic children [3]. In addition, a recent study demonstrated a decline in MIF levels during an experimental malaria infection using healthy European volunteers [4]. These studies have suggested a protective role for MIF during malaria. However, a few studies have reported an increase in MIF levels during malaria infections [2,5,6]. Elevated MIF levels were observed in malaria infected Zambian children compared to uninfected children [2]. Pregnant women with placental malaria infection demonstrated significantly higher levels of MIF in the placental intervillous blood compared to uninfected pregnant women [5,6].

The role of circulating MIF in the pathogenesis of cerebral malaria (CM) and its outcome has not been investigated. CM is a life-threatening complication of falciparum malaria; approximately 20% to 30% of CM patients die despite anti-malarial treatment and some survivors develop neurological complications [7]. It is not known why subsets of CM patients develop fatal complications and it is hoped that a better understanding of this process can lead to development of adjunct therapies that can improve treatment outcomes and aid in the identification of biomarkers associated with severe outcomes. Recent studies have implicated several factors involved in the host inflammatory pathway in the pathogenesis associated with CM [8-11]. In our own recent study with this same cohort of subjects, we observed that Interferon inducible protein 10 (IP-10), soluble tumor necrosis factor receptor 2 (sTNF-R2) and sFas were independently associated with increased risk of CM-associated mortality and that CM non-survivors had significantly lower level of the neuroprotective factor vascular endothelia growth factor (VEGF) when compared to other groups [10]. As a follow up to this study and given the role of MIF in sepsis associated death and other neurological disorders, we hypothesized that elevated levels of MIF may be involved in CM-associated death.

## Methods

For this investigation, plasma samples obtained from a well characterized prospective cohort study of malaria conducted in Jabalpur, India were utilized. This study area is a low endemic area with seasonal transmission of malaria. The study details were recently reported elsewhere [10]. This cohort had three groups of patients, namely: healthy controls (HC) who did not have malaria or other febrile illness, mild malaria (MM) patients who had fever with *P. falciparum *parasitemia of <25,000 parasites/μl of blood (detected microscopically from blood smears) and no evidence of impaired consciousness, seizures, and no past history of mental illness, meningitis, or accidental head injury, and cerebral malaria (CM) patients who met the World Health Organization's criteria for CM [12] with a Glasgow coma score of ≤8, positive blood smear for *P. falciparum *and no other clinically evident cause of impaired consciousness. Peripheral blood samples were collected from the subjects after informed consent was obtained and plasma samples were stored at -80°C until used for experiments. A total of 138 samples were available for this investigation of which 23 were from HC, 47 were from MM patients, 54 were from CM survivors (CMS) and 14 were from CM non-survivors (CMNS). Plasma MIF levels were determined using standard ELISA methods as described previously [6]. Pairwise comparisons of MIF levels in the four groups were performed through the Wilcoxon rank sum test. Logistic regression was used for the CM patients to determine if there was an association with fatal outcome and log MIF levels. Multinomial logistic regression was used for all patients to determine if there was an association with patient category (HC, MM, CMS, and CMNS) and log MIF levels, using HC as the reference group. Significance for the pairwise comparisons was assessed at the Bonferroni corrected alpha level of 0.008, and significance for the logistic regressions was assessed at an alpha level of 0.05.

## Results

**Figure 1:**
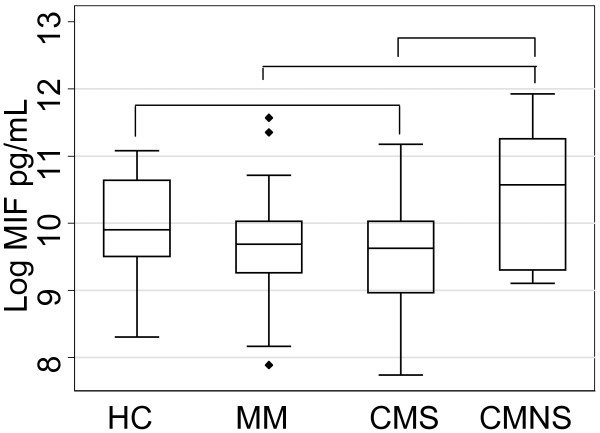
**Peripheral blood MIF levels in different groups of patients**. The log median values of plasma levels of MIF in healthy controls (HC, n = 23), mild malaria patients (MM, n = 47), cerebral malaria survivors (CMS, n = 54) and cerebral malaria non-survivors (CMNS, n = 14) are plotted as box plot with 25th- and 75th-percentile values represented by the bottom and top edges of boxes. Small filled diamonds indicate values that fall outside of the error bars. Differences were not significant after Bonferroni correction at alpha 0.008.

**Figure 2:**
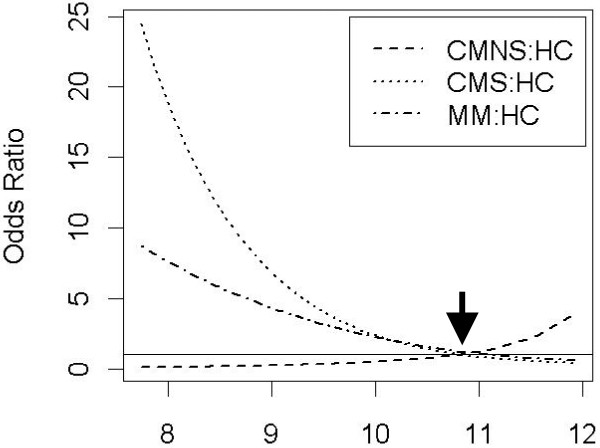
**MIF levels are associated with different disease outcomes**. The results from a multinomial logistic regression analysis are reported. The odds ratios of disease outcomes were plotted against log MIF levels. The different lines represent comparisons of the disease category to the reference healthy control (HC) group, as indicated in the figure legend. A horizontal reference line was drawn at odds ratio of 1 at which point there is a difference between a harmful and a protective factor. It is noted that the odds ratios in each of these three comparisons cross the null value of 1 at a log MIF value of approximately 10.8 (arrow).

The highest median MIF level was in the CMNS group, followed by HC, MM, and CMS, respectively (Figure 1). None of the pairwise comparisons showed any significant differences between the groups at the Bonferroni corrected alpha level of 0.008 (data not shown). The logistic regression on CM patients showed a significant association between fatal outcome and log MIF levels, with increasing log MIF levels increasing the odds of fatal outcome (odds ratio 4.0; 95%CI 1.6, 9.8; p = 0.003). The multinomial logistic regression on all patients showed that log MIF levels were significantly associated with patient category (p = 0.004). The odds ratios show that the odds of being a CMS patient compared to HC significantly decrease as log MIF increases (OR estimate 0.5; 95% CI (0.2, 0.9)). However, the odds of being a MM patient or CMNS patient compared to HC does not significantly change as log MIF increases (MM OR estimate 0.6; 95% CI (0.3, 1.1); CMNS OR estimate 2.1; 95% CI (0.9, 5.3), Figure 2).

Figure 2 is a plot of log MIF levels versus odds ratio for each patient group compared to the reference group (HC). This plot illustrates that the odds of CM or MM compared to HC decrease as log MIF increases, while the odds of CMNS compared to HC increase as log MIF increases (this is supported by the odds ratio point estimates). Furthermore, it is noted that the odds ratios in each of these three comparisons cross the null value of 1 at a log MIF value of approximately 10.8 (indicated with an arrow in Figure 2). This indicates that for log MIF values less than 10.8 a patient is more likely to belong to the CM or MM group than the HC group, and for log MIF values greater than 10.8 the patient is less likely to belong to the CM or MM group than the HC group. Conversely, for log MIF values less than 10.8 a patient is less likely to belong to the CMNS group than the HC group, and for log MIF values greater than 10.8 the patient is more likely to belong to the CMNS group than the HC group.

## Discussion

Several studies have demonstrated that elevated MIF levels were associated with inflammatory neurological disorders such as neuron-Behcet's disease and different forms of multiple sclerosis (reviewed in [1]). In a recent study, high plasma MIF levels were shown to be associated with early death in patients with severe sepsis [13]. Collectively, these findings are consistent with our hypothesis that elevated MIF levels in CM patients may be a risk factor for mortality. However, high MIF levels alone may not explain the CM-associated fatality in CMNS. It is plausible to postulate that the role of MIF in fatal CM outcome is in concert with other host factors. Indeed, host factors involved in inflammatory pathway such as TNF-α and IP-10 have been implicated in the CM associated mortality [8,10]. Since MIF up regulates the proinflammatory responses of both T- lymphocytes and macrophages, we speculate that it may act in concert with these proinflammatory factors in inducing fatal complications in CM patients. It is slightly counterintuitive that both logistic regression and multinomial logistic regression showed significant associations with log MIF levels and outcome or patient category, while the pairwise comparisons did not show significant differences in median MIF level between the patient groups. This could be due to the conservative nature of the Bonferroni correction which reduces the power to find significant differences if they do exist. However, further large scale studies are needed to confirm our findings and to investigate the potential mechanisms involved.

The observation from this study that MM and CMS patients had low MIF levels compared to HC may suggest a protective role of MIF against malaria and is consistent with previous studies that demonstrated significantly reduced circulating MIF levels in children with acute malaria infection compared to matched healthy controls [3]. Although the reasons for the down regulation of MIF in malaria patients is not well understood, a recent study suggest that this may be due to the decrease in the number of circulating lymphocytes observed during malaria infection [4]. Unlike in CMNS patients, higher levels of MIF appear to confer protective advantage to severe malaria anemia patients [14]. It has been reported that patients with an inherent ability to produce higher MIF levels are more likely to be protected from severe malaria anemia [14].

Results from this study suggest a dual role for MIF during malaria infection: a protective and pathological role. This duality in MIF functions has been suggested previously using in vitro studies (reviewed in [1,15]). However, the mechanisms involved in MIF-associated diseases are still incompletely understood and points to the fact that little is known about the role of this factor in cerebral malaria patients.

## Conclusion

Overall, these studies suggest that MIF may play a role in the fatal outcomes associated with CM. However, further studies are needed to better understand the role of MIF in malaria pathogenesis

## Competing interests

The authors declare that they have no competing interests.

## Authors' contributions

VJ: Participated in the cohort study design, enrolled patients, collected clinical and epidemiologic data and biological samples and editing of the manuscript. SM: Participated in statistical analysis of the data and editing of the manuscript. ACN: Participated in designing the cohort, enrollment of subjects, clinical evaluation and editing of the manuscript. APD: Participated in planning of the study, supervision of the study in India and editing of the manuscript. JKS: Participated in the cohort study design, planning of experiments and assisted in the editing of the manuscript. VU: Participated in the study design, data analysis, and contributed to manuscript writing and editing. NS: Participated in the cohort study design, patient recruitment, planning of the experiments and editing of the manuscript. NWL: Participated in the study design, performed the experiments and data analysis and wrote the manuscript. All authors read and approved the manuscript
